# Bioprinting salivary gland models and their regenerative applications

**DOI:** 10.1038/s41405-024-00219-2

**Published:** 2024-05-30

**Authors:** Jutapak Klangprapan, Glauco R. Souza, João N. Ferreira

**Affiliations:** 1 Avatar Biotechnologies for Oral Health and Healthy Longevity Research Unit, Faculty of Dentistry, Chulalongkorn University, 34 Henri-Dunant Road, Pathumwan, Bangkok 10330 Thailand; 2 Greiner Bio-one North America Inc., 4238 Capital Drive, Monroe, NC 28110 USA

**Keywords:** Dentistry, Oral cancer

## Abstract

**Objective:**

Salivary gland (SG) hypofunction is a common clinical condition arising from radiotherapy to suppress head and neck cancers. The radiation often destroys the SG secretory acini, and glands are left with limited regenerative potential. Due to the complex architecture of SG acini and ducts, three-dimensional (3D) bioprinting platforms have emerged to spatially define these in vitro epithelial units and develop mini-organs or organoids for regeneration. Due to the limited body of evidence, this comprehensive review highlights the advantages and challenges of bioprinting platforms for SG regeneration.

**Methods:**

SG microtissue engineering strategies such as magnetic 3D bioassembly of cells and microfluidic coaxial 3D bioprinting of cell-laden microfibers and microtubes have been proposed to replace the damaged acinar units, avoid the use of xenogeneic matrices (like Matrigel), and restore salivary flow.

**Results:**

Replacing the SG damaged organ is challenging due to its complex architecture, which combines a ductal network with acinar epithelial units to facilitate a unidirectional flow of saliva. Our research group was the first to develop 3D bioassembly SG epithelial functional organoids with innervation to respond to both cholinergic and adrenergic stimulation. More recently, microtissue engineering using coaxial 3D bioprinting of hydrogel microfibers and microtubes could also supported the formation of viable epithelial units. Both bioprinting approaches could overcome the need for Matrigel by facilitating the assembly of adult stem cells, such as human dental pulp stem cells, and primary SG cells into micro-sized 3D constructs able to produce their own matrix and self-organize into micro-modular tissue clusters with lumenized areas. Furthermore, extracellular vesicle (EV) therapies from organoid-derived secretome were also designed and validated ex vivo for SG regeneration after radiation damage.

**Conclusion:**

Magnetic 3D bioassembly and microfluidic coaxial bioprinting platforms have the potential to create SG mini-organs for regenerative applications via organoid transplantation or organoid-derived EV therapies.

## Introduction

Salivary glands (SGs) play a crucial role in the oral cavity, facilitating essential functions such as lubrication, enzymatic digestion, and bacteriostasis through saliva secretion. Dysfunction in the SGs, triggered by factors like aging, polypharmacy side effects, autoimmune conditions, and anti-cancer therapies, can impact speech, digestion, and oral health. For instance, radiation therapy for head and neck squamous cell carcinoma (HNSCC) often damages SG, causing hyposalivation and xerostomia (dry mouth). Current treatments provide only temporary relief, posing a significant challenge for the 1.1 million new head and neck cancer cases (HNSCC included) that are diagnosed annually worldwide [1].

To address this issue, a biopsy of healthy SG tissue before radiation therapy could enable the ex vivo development of a tissue-engineered substitute for autologous re-implantation, aiming to restore salivary function. However, SGs, with their complex structure and multiple cell types, present challenges for replacement [2, 3]. Many existing SG engineering approaches involve encapsulating SG-derived epithelial cells in hydrogel matrices; yet, spatial control is limited, especially when mimicking the branched architecture of native SG [4–7]. Fine epithelial layers of ducts and acini, being exceptionally thin, are challenging to recreate synthetically. In 2018, our research group developed for the first time innervated three-dimensional culture (3D) organ building blocks of the SG, spheroids or organoids, using magnetic bioassembly and levitation of adult stem cells from the dental pulp and SG primary stem/progenitor cells, respectively [8, 9]. Earlier, we have provided the SG research field a very comprehensive review of our magnetic 3D bioassembly (bioprinting and levitation) strategies and a state-of-the-art summary of the current regenerative therapies for the salivary gland up to the year 2022 [10]. However, several reports of novel 3D approaches for regenerative medicine and SG tissue engineering were published since then, hence, this review is pertinent [11–17]. Recently, our group was able to effectively expand SG primary cells in the short-term with high proliferative rate when using plant molecular farming-derived cues in combination with hyaluronic acid/alginate hydrogels [13] or cell sheets on top of decellularized extracellular matrix (dECM) bioassembled in porous polymers [11]. Efforts have been made by our team to modify SG ECM properties and improve the cell-matrix interface, impacting cluster morphology and the distribution of key proteins. Yet, achieving spatial control in 3D organ building blocks remains a hurdle, particularly in replicating the intricate structure of SG. In the evolving field of 3D cell culture and bioprinting, significant progress has been made in the formulation of biomaterials at a mesoscale, typical of cell aggregates or tissues at early development [8, 9, 17, 18]. These mesoscale biomaterials can serve as scaffolds for in vitro production of microtissues, organoids, spheroids or assembloids capable of performing key physiological functions resembling those of native tissues. A “modular” approach to microtissue engineering involves arranging distinct hydrogel compartments, loaded with different types of cells, into a biomimetic scaffold. This strategy offers well-defined initial conditions for tissue growth and maturation, including cell proliferation and differentiation into functional tissue. More importantly, compartmentalization enables not only 3D cell patterning but also biopolymer spatial patterning, allowing for the imposition of varying physicochemical cues, such as local gradients in matrix stiffness or molecular protein content. However, these strategies may not be enough to avoid a central necrotic core in these tissue constructs if matrices or cells are too compact or well-packed to allow for proper nutrient mass transfer.

Since 2018, our research group has been developing magnetic 3D bioassembly platforms to produce micro-modular cellular clusters to synthesize their own ECM (internally) and meet the needs of microtissue engineering [8, 9, 11, 13, 14, 19]. Our group has ongoing efforts to fabricate SG organoids through magnetic 3D bioassembly platforms to create matrix-free, reproducible, scalable and functional 3D mini-organ prototypes for regenerative studies combining cells with dECM and plant molecular farming cues focused on SG transplantation and EV therapies for SG regeneration [11, 13, 20]. This review will go over the relevant benefits and challenges of these new platforms.

## Innovation paths from 2D to 3D SG culture

**Fig. 1:**
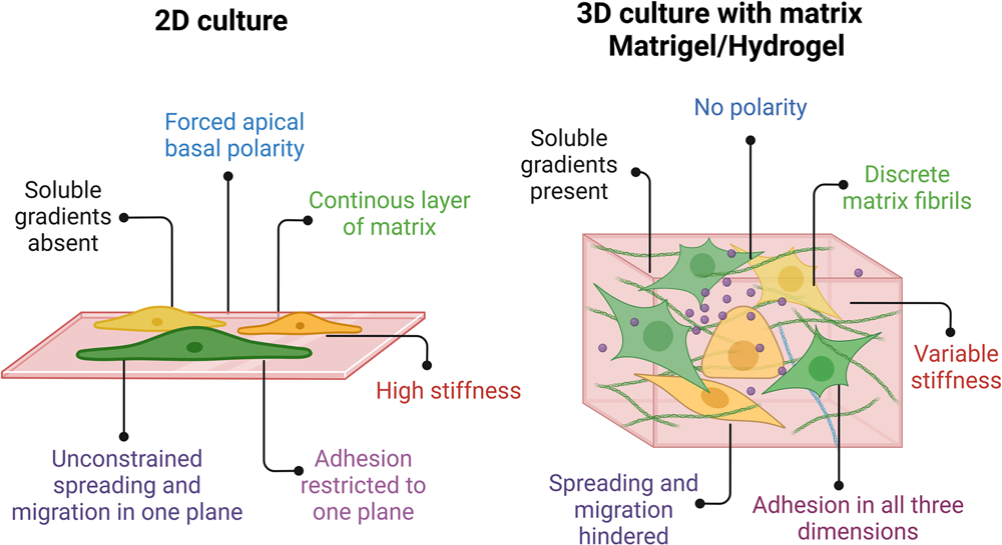
Comparison of 2D and 3D cell culture. This highlights the differences in cell behavior and constraints between 2D and 3D environments, embedded in Matrigel/hydrogels or other ECM proteins. Created with BioRender.com

Traditional monolayer or two-dimensional (2D) cell cultures have been utilized as in vitro models for cell biology research and drug discovery and screening for several decades [21]. In this system, cells are in contact with a nutrient-rich medium and grown as a monolayer on glass or plastic dishes. 2D cell culture provides an easy approach to study cell behavior through imaging or gene expression profiling. Its efficiency allows for high-throughput screening in drug discovery [22]. However, 2D models have limitations especially since they do not accurately recapitulate the natural architecture and unit structure of tissue. In 2D, cell–cell and cell–ECM interactions do not represent the in vivo reality, and these are crucial for cell behaviors, like cell proliferation, differentiation, migration, or morphogenesis [23, 24]. In vivo microenvironments in 2D are inaccurate due to factors like monolayer growth in rigid platforms [24], uneven nutrient distribution, and dynamic spatial gradients affecting cell behavior [25]. This results in the loss of diverse cellular phenotypes, affecting function, internal structure organization, secretion, and signaling. Adherent cells lose polarity, altering their response to phenomena like apoptosis, and disrupted external interactions disrupt their internal structure organization [26, 27] (Fig. 1).

Organoids, categorized as three-dimensional (3D) mini-organs, created from primary cells or stem cells in a culture dish in the presence of signaling cues specific to that particular organ. To enhance the physiological microenvironment and enable the 3D arrangement of cells, researchers have developed 3D culture techniques. These techniques can be broadly categorized into three types: (1) those using nonadhesive substrates that promote cell–cell interactions, resulting in the aggregation of cells into spheroids (without external hydrogel support), (2) those embedding cells within an ECM-like hydrogel matrix for providing external support, (3) those that involve tagging cells with nanoparticles to render them magnetic and then assemble such cells into a specific shape/morphology via magnetic fields (dot, disc, or ring-like magnets) to force cell-cell interactions that ultimately lead to endogenous ECM production in culture (hence, no external substrates, hydrogels or ECM are needed).

SG organoids are often derived from primary adult SG stem/progenitor cells or pluripotent stem cells (PSCs) using 3D matrix [28, 29]. A 3D matrix is crucial for organoid development, mimicking ECM for cell attachment, proliferation, and differentiation. Cell spheroids can be formed using various 3D culture techniques including rotating culture vessels, hanging drop, spontaneous cell aggregation and magnetic 3D bioassembly [30, 31]. SG primary cells can form salivary functional spheroids in serum-free culture, while preserving native ECM and expressing acinar cells, epithelial polarization, and tight junction proteins. Additionally, salivary spheroids cultured in ECM-derived 3D matrices can maintaining the structural integrity for 10 days or more [32]. However, after 10 days of spheroid culture, cellular apoptosis can occur due to uncontrollable increase in size, lower oxygen diffusion rate and nutrient limitations. Hence, controlling the size of spheroids is challenging, especially when high cell density is needed, often resulting in cell death in the central core [33].

## 3D scaffold materials for SG tissue engineering

**Fig. 2:**
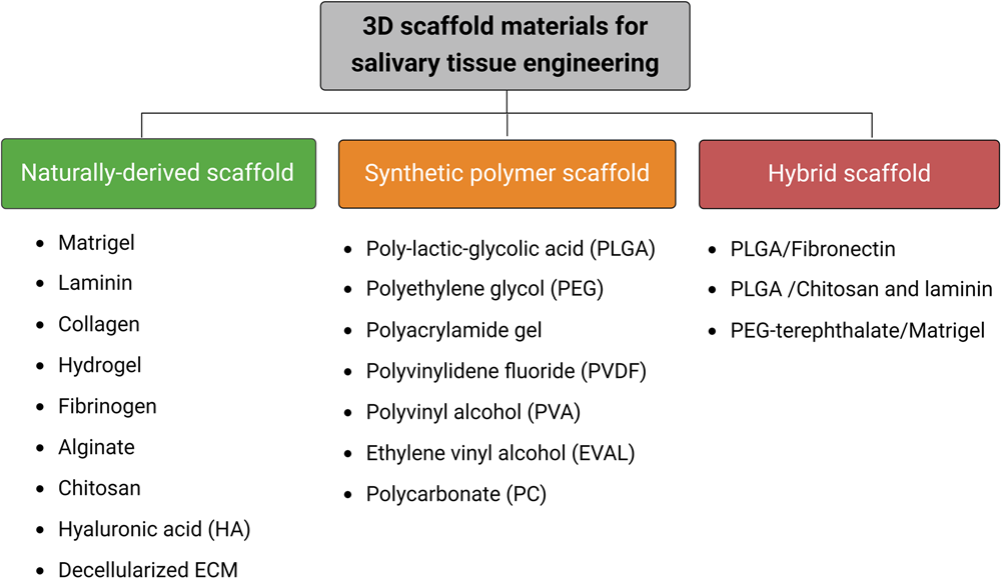
Classification of biomaterials for 3D scaffold preparation in salivary tissue engineering. The diagram illustrates three main categories of biomaterials used in the construction of 3D scaffolds: naturally-derived scaffold biomaterials, synthetic scaffold polymers, and hybrid scaffold materials. Created with BioRender.com

3D scaffold materials play a crucial role in replicating the mechanical, physiochemical, and biological characteristics of SG tissue engineering. They have been classified into three groups: naturally-derived scaffold biomaterials, synthetic scaffold polymers, and hybrid scaffold materials (Fig. 2). The 3D scaffolds and cell sources potentially used for salivary tissue engineering were listed in Table 1.

**Table 1:** Potential 3D scaffolds and cell sources for salivary gland tissue engineering.

3D scaffolds	Cell sources	Ref.
Matrigel	Human SMG cell lines	[36]
Matrigel	Rat PG cells	[37]
Laminin peptides conjugated Fibrin hydrogel	Rat PG cells	[7, 41]
Alginate-hydrogel	Human salivary stem/progenitor cells	[17]
Human placenta BME	Human SG cells	[42]
BME-derived peptides modified-hyaluronate hydrogel	Human salivary stem/progenitor cells	[49]
Decellularized SMG	Rat SMG cells	[43]
Polyethylene glycol hydrogel (PEG)	Mouse SMG cells	[6]
Poly-lactic-co-glycolic acid (PLGA)	Rat PG cells	[5]
Matrigel-coated PEG	Porcine PG cells	[57]
Chitosan + laminin - coated PLGA	Mouse SMG cell lines	[61]

*SMG* submandibular gland, *PG* parotid gland, *BME* basement membrane, *SG* salivary gland

### Naturally-derived scaffold biomaterials

Matrigel, a solid hydrogel that mimics the native ECM, is derived from mouse sarcoma cell cultures and is composed of ECM proteins, including collagen IV, laminin, heparan sulfate, and growth factors (to a certain extent), which support SG branching morphogenesis [34]. Recent advancements in 3D cell culture have integrated these approaches using microgel (hydrogel-based) scaffolds which offer controlled confinement and a highly biomimetic local 3D microenvironment, enabling the generation of reproducible and biologically relevant microtissues [4, 35]. Matrigel, a widely used biomaterial in tissue engineering, supports cell attachment and promotes differentiation in vitro [36–38]. Despite, its animal origin, lot-to-lot variation and challenges due to the existence of many elements that can modulate cell behavior, it is not suitable for clinical translation. Laminin, a key component of the basement membrane (BM) has been studied for identifying the relationship between specific ECM constituents and cell activity. Laminin is essential for SG development and morphogenesis [39] but the entire laminin-1 sequence can lead to adverse effects, including degradation, tumorigenesis, and adverse immune responses making it unsuitable for clinical applications [40]. Interestingly, peptides derived from ECM can support cell-matrix attachment and adhesion within 3D scaffolds. Certain peptides derived from laminin have the ability to enhance the formation of SG spheroids, branching morphogenesis, and promote SG function [7, 41]. Notably, human fibronectin-based hydrogels, with placental BM extracts can generate polarized salivary acinar-like structures [42]. Additionally, the dECM from SG can be utilized as a naturally-derived biomaterial. An earlier study using whole dECM from rat SG demonstrated the ability to support the adhesion of primary SG cells, facilitating the formation of SG-like tissues [43]. Also, dECM from human submandibular gland (SMG) tissues has been employed as a substrate for cultivating human epithelial cells as well as fibroblasts [34]. However, biomaterials from animal or human tissues lack reproducibility, tunability, and may elevate the risk of tumorigenic or immunogenic reactions [44].

Hydrogels are networks of hydrophilic polymers arranged in 3D, formed through chemical or physical links, incorporating natural, synthetic, or hybrid polymers. Natural polymer-based hydrogels are a favorite choice because of their similarity to human ECM and bioactivity. Recent study suggests hyaluronate (HA)-based hydrogels as potential natural biomaterials for generating SG organoids [44]. The enhanced HA-based hydrogels, modified with basement membrane-derived peptides from perlecan and laminin bioactive domains, promoted increased proliferation of SG acinar-like cells, accompanied by lumen formation and increased α-amylase secretion [45–47]. After encapsulation in HA-hydrogels, human SG primary cells were successfully transplanted into an irradiated SG model, resulting in the secretion of α-amylase intra-orally [48]. Moreover, a modified fibrin hydrogel containing A99 and YIGSR peptides from laminin-1 protein was assessed for cell migration and adhesion [41]. This model promoted lumen formation in SG spheroids and enhanced the expression of the cell adherens junction protein E-cadherin and tight junction protein ZO-1 in acinar epithelial cells at the surgical site, 8 days after transplantation.

Currently, alginate-based hydrogel has been utilized for SG tissue engineering, creating microfiber or microtube structures [17]. Yin et al. created thin salivary epithelia using this hydrogel in two printing modes: solid fibers for branching SG structures and hollow tubes with 45–80 μm wall thicknesses, featuring a sacrificial liquid core and thin hydrogel walls. This coaxial microfluidics allows for the fabrication of thin features, such as cell-laden microtubes, ensuring cell viability and reproducibility, while retaining the capability to print larger hydrogel structures at the cm- and mm-scales [17]. Furthermore, hydrogels modified with arginine-glycine-aspartic acid and scaffolds based on chitin/chitosan promoted the expansion of SMG buds, branching and facilitated essential ECM production [49, 50]. This demonstration of nano-scale technologies in SG engineering opens up new avenues for exploring the spatial customization of ECM components, intercellular communication, and specific cytokine delivery.

### Synthetic polymers scaffolds

Scaffolds made from synthetic polymers are extensively utilized in tissue engineering, as they provide customized mechanical and physicochemical properties conducive to cell growth and differentiation [51]. These can provides a scalable, xenogenic-free environment that can be tailored for desired outcomes, offering a viable alternative to natural sources like Matrigel. This will ensure reproducible results and large-scale production without limitations [52]. Polyethylene glycol (PEG) as well as poly-lactic-co-glycolic acid (PLGA) are commonly utilized as 3D scaffolds for SG tissue engineering [5, 6]. Electrospun-PLGA nanofibers can mimic the ECM nanoscale structure for SG epithelial cells, promoting polarization and differentiation through water channel protein expression [5]. A report has found that methacrylate-based polymerization in PEG-based hydrogels decreases viability of SMG cells, while thiol-ene polymerization is more effective for encapsulating these cells [6]. However, the encapsulation of individual cells in hydrogels does not lead to the formation of the SG structure. Enhancing cell viability, promoting proliferation, and preserving cell-cell contacts can be achieved by encapsulating pre-assembled multicellular spheroids in these hydrogels [6]. Notably, polyacrylamide gels, known for their physiological compliance, have been used to evaluate substrate modulus on SMG regeneration. These gels facilitate higher branching morphogenesis in SG compared to stiffer gels [53]. Additionally, polyvinylidene fluoride (PVDF), when compared to other synthetic biomaterials such as polycarbonate (PC), polyvinyl alcohol (PVA) and ethylene vinyl alcohol (EVAL) enhanced branching morphogenesis in serum-free cultures [54, 55].

### Hybrid scaffold materials

Hybrid scaffold materials refer to those that contain both natural and synthetic polymers which were produced to enhance cell attachment and differentiation. SG cells demonstrate adhesion to polymer disks coated with ECM peptides and exhibit similar behavior on both polyglycolic acid (PGA) and poly-L-lactic acid (PLLA) substrates [56]. A report found that Matrigel coating PEG-terephthalate/poly(butylene terephthalate) scaffolds enhanced the growth and morphology of SG epithelial cells [57], while laminin- and chitosan-coated PLGA nanofibers promoted the proliferation of SG epithelial cells [58]. Despite their favorable structural stability and mechanical properties, synthetic polymers may have poor biocompatibility, cytotoxic degradation products, and inadequate bioactivity. Natural polymers, while beneficial for biocompatibility and biodegradation, often have poor mechanical properties even after cross-linking. This makes them difficult to use for reconstructing tissues requiring high mechanical loading [59, 60].

Those ECM-based strategies offer promising biomimetic 3D scaffolds for generating SG organoids from stem/progenitor cells in combination with different natural or synthetic polymers. They provide 3D growth and differentiation into a functional organoid/tissue, certainly useful for studying the in vivo physical environment [61–63]. However, Matrigel hydrogels have challenges in terms of reproducibility due to biochemical variations and clinical translation. The use of these ECM platforms is costly due to their long biofabrication process, inconsistent cellular aggregates, and time-consuming analysis, and 3D cell culture protocols can take 9–12 days for the formation of spheroids [18, 61].

## Magnetic 3D bioassembly platform for SG organoid biofabrication

**Fig. 3:**
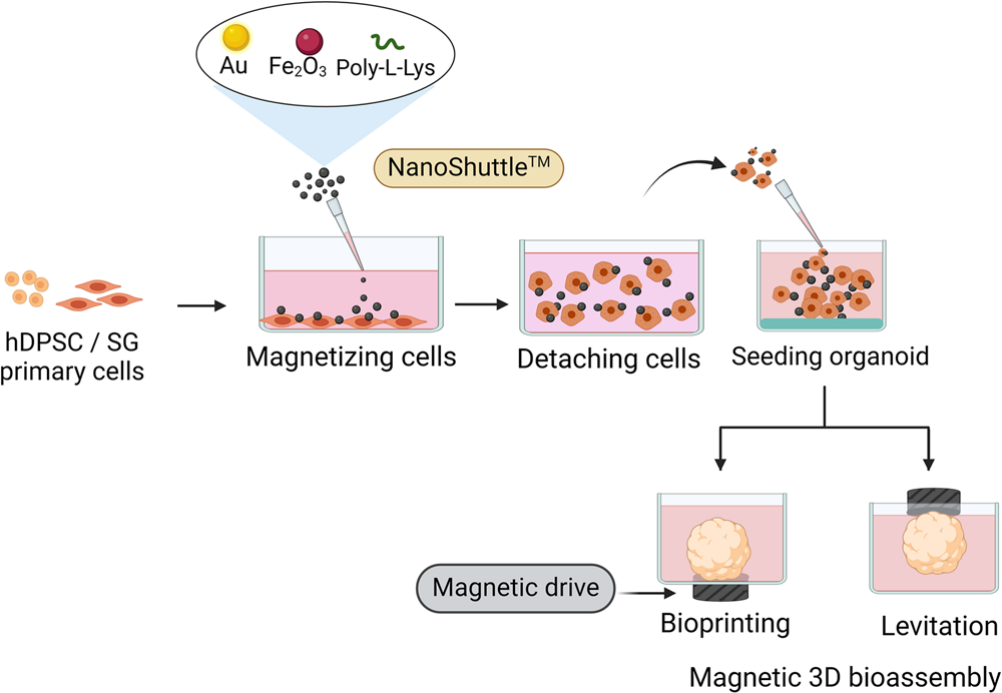
SG organoid biofabrication workflow utilizing two different magnetic 3D bioassembly platforms. Human dental pulp stem cells (hDPSC) or salivary gland (SG) primary cells were magnetized with magnetic nanoparticle (MNP), also referred to as Nanoshuttle. Subsequently, the cells were detached and seeded into an ultra-low attachment 96-well plate. Bioprinting refers to when a plate with cells is placed on top of a magnetic field (with magnet dots), and levitation is when the magnets are positioned on top of the plate, both of which induced cell aggregation. Created with BioRender.com

Magnetic 3D bioassembly is a simple platform to create a scaffold/substrate-free 3D culture within 24 h depending on the cell type and the concentration of magnetic nanoparticles (MNP), allowing for consistent organoid formation, media handling and replacement, and high-throughput imaging using confocal microscopy and immunofluorescence techniques [64–66]. In this platform, cells in a monolayer are magnetized with MNP comprising iron oxide, gold, and poly-L-lysine (size ~50 nm) through electrostatic interactions with the plasma membrane [65]. Subsequently, the cells are detached from plastic surfaces, suspended in culture media and manipulated with external magnetic forces (dots on magnetic drives). There are two methods depending on the positioning of the magnetic drive: (1) when the drive is placed under the culture plate, the approach is called “magnetic 3D bioassembly (M3DB)” and (2) when the drive is placed on top of the well plate, it is termed “magnetic 3D levitation (M3DL)” (Fig. 3). This latter concentrates the SG cells at the air-liquid interface for better nutrient reperfusion. Both are employed to accelerate cell aggregation, promote tight junction formation, and induce the epithelial spheroids [67, 68]. The SG organoid biofabrication workflow utilizing magnetic 3D bioassembly platform is displayed in Fig. 2. The resulting 3D cellular constructs are dense, spatially organized, and capable of synthesizing ECM without engineered substrates or scaffolds or, relying on cell-derived bio-matrices for tissue morphogenesis and cell-matrix interactions. After that, these 3D spheroids/organoids can be analyzed using assays like cytotoxicity, immunohistochemistry, western blotting, and other biochemical techniques on a similar fashion as the regular 2D/3D culture systems [67]. More surprisingly, the MNPs used in these platforms (M3DB and M3DL) support cellular metabolism and proliferation without inducing proinflammatory and oxidative stress [65]. They have demonstrated biocompatibility and elicited a negligible immune response after transplantation [69]. Some articles have reported that gold and iron oxide nanoparticles can be toxic to cells, causing cell membrane disruption, DNA damage, oxidative stress, and impaired mitochondrial function [15, 70]. However, the extent of toxicity depends on factors such as concentration and cell uptake, as well as nanoparticle aggregation in biological media and serum. Despite intracellular uptake by breast cancer cells (BT-474 and MDA-MB-231), these nanoparticles did not exhibit significant (<10%) cytotoxicity at concentrations up to 100 μg/ml [71]. Therefore, optimizing nanoparticles for specific applications is essential.

Our research team has successfully produced organoids resembling secretory exocrine organs using both M3DB and M3DL [8, 9, 20, 72]. These bioassembly systems can incorporate various human SG cell types, including epithelial, myoepithelial, neuronal and endothelial cells, into organotypic systems [9]. Our study demonstrated this platform for creating bio-functional SG organoids from human dental pulp stem cells (hDPSC). These cells undergo expansion and specialized differentiation into secretory and innervated SG-like epithelial organoids through cultivation with epithelial and neurogenic differentiated media [8]. Also, hDPSC organoids express specific epithelial markers and display SG secretory functions under muscarinic and adrenergic stimulations. Fibroblast growth factor 10 (FGF10), a crucial signaling cue for SG morphogenesis [73], was supplemented in media to promote hDPSC differentiation towards epithelial and neuronal tissue biofabrication [8]. SG organoids via M3DB showed SG-like epithelia with neuro-functional properties, secreting α-amylase and intracellular calcium mobilization. A recent study utilized porcine primary SG and lacrimal grand (LG) cells on the M3DB platform to create functional craniofacial exocrine gland organoids for in vitro aging models. These organoids expressed functional acinar and ductal units with epithelial progenitors, and successfully induced cellular senescence to mimic the native SG and LG hypofunction representing dry eyes and dry mouth [74]. Notably, the transplantation of organoids rescued both the epithelial bud and neuronal components of ex vivo irradiated SG. Curiously, there was a integration between the neuronal networks of the ex vivo irradiated SG and SG-like organoids [8]. In addition, our group has developed magnetically assembled dECM constructs from porcine SG primary cells. These dECM constructs support primary SG cell proliferation, tethering, and differentiation compared to conventional culture plastic dishes and surfaces coated with basement membrane extract (also called BME) [11].

Later on, the M3DL platform was developed to overcome technical shortcomings such as poor light penetration and nutrient diffusion in the center of organoids [68]. The study used porcine SG primary cells to create in vitro SG-like organoids using M3DL. This organoid had positive SG markers from various cellular SG compartments, including adherens junctions (EpCAM, E-cadherin), ductal epithelial and myoepithelial (Cytokeratin 14 and α-smooth muscle actin), and neuronal (β3-tubulin and vesicular acetylcholine transferase). They also exhibited intracellular calcium activity and *α*-amylase secretion in response to cholinergic stimulation [10]. In the process of SG organoid development, establishing apicobasal polarity in epithelial cells and forming branched lumenized ducts are crucial for directing salivary flow and facilitating saliva production. Achieving these epithelial polarity properties in SG organoids or mini-glands has proven challenging [46]. Nonetheless, the application of these bioassembly strategies has demonstrated promise in in vivo rodent models through the use of magnets [69]. In this specific in vivo study, the magnetized stem cells were biocompatible and effectively targeted.

## EV generation from bioassemble SG organoids

Extracellular vesicles (EV) are emerging as nano-based therapeutic approaches in regenerative medicine due to their ability to target specific targets, such as cell proliferation, immunomodulation, and angiogenesis [75–77]. The use of EV combined with biomaterials and bioengineering techniques can enhance tissue repair and facilitate wound healing. Notably, EV lack immune rejection and cytotoxicity, are promptly preserved, and can maintain their bioactive properties for long-term storage towards future SG tissue engineering applications [12, 76]. Furthermore, EV can be accurately quantified and produced in large quantities during cell line in vitro expansion without invasive extraction procedures [78]. This not only leads to cost reduction but also results in shorter therapy durations. The presence of EV in body fluids such as blood, breast milk, and saliva are noteworthy [79–81]. These transport a cargo of proteins, RNA, DNA and lipids, facilitating their exchange or transport between cells [82]. In addition, EV have proteins that shed from the cell membrane as well as intracellular proteins. These proteins play a regulatory role in cell-to-ECM interactions and cell-to-cell communications [83]. Remarkably, EV exert control over cellular processes and can traverse tissue barriers [84]. This characteristic makes them a highly sought-after targeted structure in adult stem cells for the discovery of novel biomolecules, particularly in endothelial and neuronal networks, thereby advancing tissue regeneration.

Mesenchymal stem cell (MSC) transplantation is a viable model for tissue regeneration; however, its application in clinical settings is intricate and time-consuming. This process involves extended culture times and relies on individual availability. Notably, MSC can release EV, including exosomes and macrovesicles, which act as paracrine cues mediating communication between MSC and target cells. This unique capability potentially mirrors the biological activity of MSC and offers an alternative to whole cell therapy [85, 86] In a recent study, EV secreted from SG-derived MSC could repair the damaged tissue and restore SG hypofunction in obstructive sialadenitis [12]. These EV exhibit anti-inflammatory effects in lipopolysaccharide-induced organoids modelling inflammation and in macrophage polarization by reducing acinar-to-ductal metaplasia. More interestingly, miRNAs from EV have been found to target and regulate certain inflammation-related pathways [12]. Furthermore, EV can be extracted from hDPSC after 24–72 h of culture in serum-free media [87, 88], promoting acinar epithelial repair in a mice model, suggesting efficacy in SG regeneration strategies [16, 84]. Dental literature has reported the functional significance of hDPSC-derived EV, indicating their ability to activate dental pulp tissue regeneration, induce stem cell differentiation [89], and promote angiogenesis [77].

**Fig. 4:**
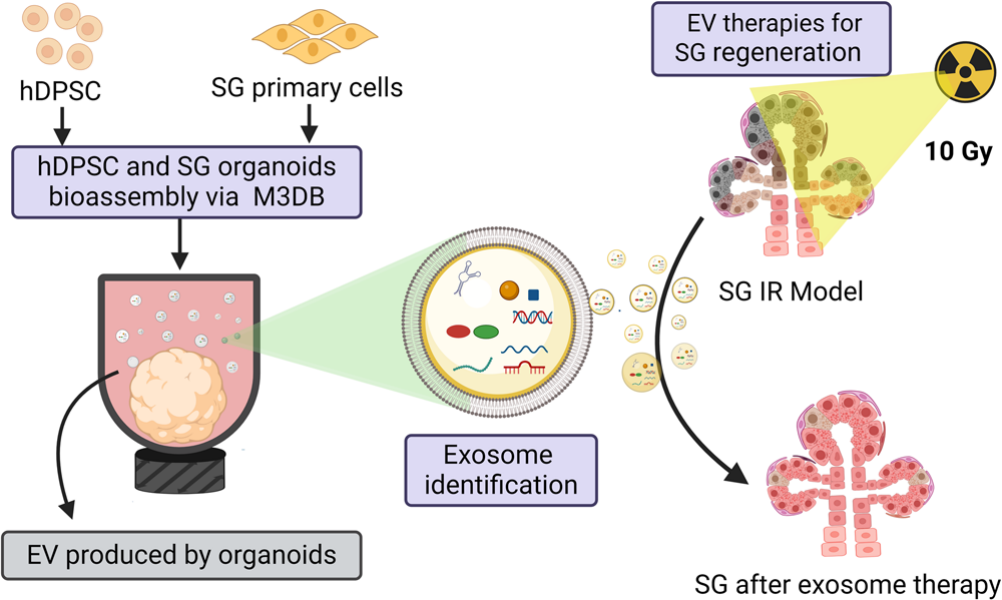
Flowchart outlining EV-based strategies for SG epithelial repair using hDPSC cultures and SG organoids prepared via M3DB. hDPSC and SG primary cells were assembled into organoids using a magnetic drive in a 96-well ultra-low attachment plate. Then, EV was extracted from conditioned media and identified as exosomes. Magnetic bioassembly of hDPSC-derived and SG organoid-derived exosomes (100% extract) was then administered into SG growth media to treat epithelial repair in irradiated (IR) SG models. Created with BioRender.com

Our previous research on SG organoids developed with the M3DB platform showed they can promote epithelial and neuronal growth in irradiated SG after transplantation [8]. These therapeutic outcomes are perhaps due to the paracrine role of the EV secreted by the mesenchymal-derived adipose tissue (ASC) [90–92]. These effects were alike the ones seen with the hDPSC-derived EV in our SG bio-printed organoid. However, the function of the EV secreted from M3DB in SG epithelial repair remains unexplored. Therefore, assessing EV from hDPSC and SG organoids in vitro is crucial for understanding SG epithelial repair and optimizing the SG organoid platform for enhanced EV paracrine cue release. Our previous study investigated the use of EV derived from hDPSC and SG organoids (fabricated from M3DB) in ex vivo irradiated SG fetal models (Fig. 4). EV from SG were consistently higher than those from hDPSC organoids. These ex vivo models have shown that EV from condition media of bioassemble SG organoids promote SG regeneration by increasing epithelial bud proliferation, SG progenitors, and neuronal growth of irradiated fetal SG. Moreover, EV from human SG organoids played a more relevant paracrine function in epithelial SG growth and repair (~60%) when compared to exosomes from hDPSC organoids (~15%) and SG organoid transplants (~25%) [20]. This report suggests that exosomes from M3DB-assembled SG organoids can improve SG epithelial damage. SG organoids demonstrate the capacity to release EV, suggesting a therapeutic potential that merits exploration in future studies.

However, the role of exosomes in in vivo SG regenerative models post-radiotherapy remains untested due to their complex biological cues. The International Society for Extracellular Vesicles or ISEV conducted a global survey for Minimal Information for Studies of Extracellular Vesicles (MISEV2023) guidelines [93], in collaboration with our research group and others. This survey was to provide an updated overview of the advantages and limitations of producing, separating, and characterizing EV from various sources, including body fluids, and solid tissues especially cell culture. Cell culture conditions significantly affect the yield, composition, and function of EV [94, 95] Cell cultures may also contain xenogeneic contaminants such as exogenous bovine RNA, EVs and proteins complexes. This contamination limits the clinical applications of EVs, as it can affect their cargo composition [96, 97] Hence, the EV purification method to obtain a high yield and purity of EV needs to be carefully considered.

## Potential therapeutic applications of organoids generated by M3DB/M3DL

**Fig. 5:**
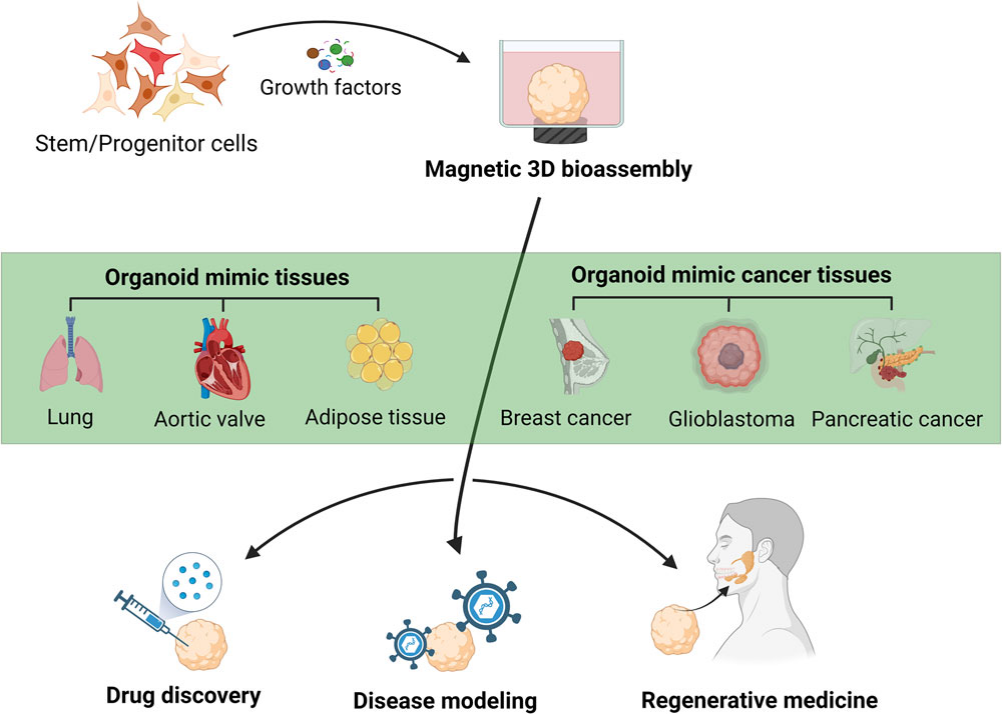
Potential therapeutic applications of organoids manufactured by M3DB. This M3DB platform has demonstrated the ability to generate organoids from mimicking various tissues, including lung, aortic valve, adipose tissue, and cancers such as breast, pancreatic and glioblastoma. These printing capabilities and prototyping make it crucial for drug discovery, disease modeling, and regenerative medicine applications. Created with BioRender.com

Radiation-induced xerostomia is a severe condition affecting HNC patients, affecting their quality of life. The SG are sensitive to radiation, causing damage to acinar cells, blood vessels, and nerves. SG have limited self-healing abilities, making xerostomia often irreversible [98, 99]. Magnetic 3D bioassembly platforms are a potential solution for bioengineer SG neotissue to restore salivary function in cases of damaged SG due to irradiation [20]. This platform offers the exquisite control and manipulation of spatial arrangements, thereby providing a level of precision and viability crucial for applications spanning drug discovery, disease modeling, and regenerative medicine [68, 100, 101] (Fig. 5).

In the present, we are investigating magnetic 3D bioassembly approaches in SG cancer organoids as a potential avenue. Remarkably, our SG cancer organoids, cultured for two days, exhibit significant expression of SG ductal epithelial markers (Cytokeratin 14), a proliferation marker (Ki67), and cell adherens junction proteins, demonstrating consistent size at 300 µm diameter and reproducibility. With this robust model in place, the next research steps involve advancing towards the testing of radiotherapy platforms, leveraging the potential of the magnetic 3D bioassembly platform to enhance our understanding and therapeutic interventions in SG cancer research. Furthermore, this 3D platform has previously demonstrated its capability to recapitulate the native extracellular matrix in various tissues for modular organ prototyping, including the lung [102], aortic valve [103], adipose tissue [66] and cancer organoids such as breast [104] pancreatic [101] and also glioblastoma [68] (Fig. 5). Organoids, mimicking adult organs in both architecture and function, emerge as invaluable tools for the comprehensive study of tissue repair mechanisms and the intricacies of human diseases.

Magnetic 3D bioassembly nanotechnology plays crucial rules in the SG regeneration field, creating scaled-up, xeno-free, and biocompatible tissue compartments for cell growth, differentiation, and biointegration. These strategies enable co-culture methods for generating SG matrices, cell-derived secretome, and microtissue compartments, and integrating the complexity of human SG components within a 3D architectural framework. In addition, they provide a platform to explore novel surgical techniques using magnetic fields, facilitating the in vivo implantation and stabilization of magnetized SG organoids or mini-glands in sites of injury [69].

## Conclusions

Magnetic 3D bioassembly platforms, gold-based MNPs modified with poly-L-lysine and iron oxide, have the potential to bioassemble SG organoids for regenerative applications via organoid transplantation or EV therapies from organoid-derived secretome. In our ongoing studies, SG cancer organoids can be generated via M3DB, and in the future, we expect to use these for high throughput in vitro screening applications with RT fractionated dose regimens. Our research team is currently developing nanoparticles that can serve as potential radiosensitizers to stop the progression of SG cancers.
